# Effect of Hot Metal Gas Forming Process on Formability and Microstructure of 6063 Aluminum Alloy Double Wave Tube

**DOI:** 10.3390/ma16031152

**Published:** 2023-01-29

**Authors:** Yong Xu, Xiu-Wen Lv, Yun Wang, Shi-Hong Zhang, Wen-Long Xie, Liang-Liang Xia, Shuai-Feng Chen

**Affiliations:** 1 Shi-Changxu Innovation Center for Advanced Materials, Institute of Metal Research, Chinese Academy of Sciences, Shenyang 110016, China; 2 School of Materials Science and Engineering, University of Science and Technology of China, Shenyang 110016, China; 3 College of Materials Science and Technology, Nanjing University of Aeronautics and Astronautics, Nanjing 210016, China; 4 College of Metallurgy and Energy, North China University of Science and Technology, Tangshan 063210, China

**Keywords:** hot metal gas forming (HMGF), 6063 aluminum alloy, formability, microstructure, dynamic recrystallization

## Abstract

The hot metal gas forming process can significantly improve the formability of a tube and is suitable for the manufacturing of parts with complex shapes. In this paper, a double wave tube component is studied. The effects of different temperatures (400 °C, 425 °C, 450 °C and 475 °C) and different pressures (1 MPa, 1.5 MPa, 2 MPa, 2.5 MPa and 3 MPa) on the formability of 6063 aluminum alloy tubes were studied. The influence of hot metal gas forming process parameters on the microstructure was analyzed. The optimal hot metal gas forming process parameters of 6063 aluminum alloy tubes were explored. The results show that the expansion rate increases with the increase in pressure. The pressure affects the deformation of the tube, which in turn has an effect on the dynamic softening of the material. The expansion rate of parts also increases with the increase in forming temperature. The increased deformation temperature is beneficial to the dynamic recrystallization of 6063, resulting in softening of the material and enhanced deformation uniformity between grains, so that the formability of the material is improved. The optimum hot metal gas forming process parameters of 6063 aluminum alloy tubes are the temperature of 475 °C and the pressure of 2.5 MPa; the maximum expansion ratio is 41.6%.

## 1. Introduction

Aluminum alloy has the characteristics of lightweight structure, high strength, good electrical conductivity and corrosion resistance, and the light tube made of aluminum alloy can meet the requirements of optimized structural design and is lightweight, so it is widely used in aerospace, transportation and other fields [1,2,3,4,5]. In order to rationalize the spatial structure of components, the structural design of tube parts is increasingly complicated, and its geometric structure is mostly characterized by an overall large expansion rate and local small features. At present, the forming technology of tube includes hydroforming [6,7], particle-assisted tube forming [8], magnetorheological elastomer forming [9], tubular channel angular pressing (TCAP) and parallel tubular-channel angular pressing (PTCAP) [10,11] and so on. However, due to the high deformation resistance and poor formability of aluminum alloy at room temperature, it is difficult to manufacture by hydroforming, which hinders further wide application [12,13].

Increasing the forming temperature can soften the tube and improve its formability to a certain extent. However, the highest temperature of liquid medium in hydroforming is only boiling temperature, which cannot provide a higher temperature as high-pressure medium for parts forming. Therefore, gas that can achieve high temperature and provide high pressure is considered as the pressure transfer medium for forming parts in high temperature. Hot metal gas forming (HMGF) was proposed as a modern plastic processing method. The parts were heated to near the recrystallization temperature, and gas with pressure was applied to shape the part [14]. The hot metal gas forming process is used to produce parts with complex shapes, especially those with large curvature and small fillet features [15,16]. This process can significantly improve the formability of parts, improve the forming efficiency of hard-to-deform tubes and reduce the weight and production cost of parts. At present, some engineering applications of HMGF process have been realized, such as special-shaped tubes and complex tube connectors for intake and exhaust systems in aircraft [17,18].

Liu et al. [19] studied wall thickness distribution uniformity and formability of parts in the process of deformation by using different bulging pressure in the temperature range of 350~450 °C, and the results showed that temperature uniformity has a great influence on the formability and microstructure of parts. Michieletto et al. [20] studied the HMGF process bulging behavior of AA6060 aluminum alloy tube used for automobiles with different forming rates and temperatures, and the research showed that the microstructure and thickness distribution of parts formed at different forming rates were not significantly different, and the increase in forming temperature would increase the sticking rate of parts. Moreover, the length of the initial blank has little influence on the forming of the parts. Rajaee et al. [21] studied the influence of temperature, forming pressure and axial feed on HMGF of 6063 aluminum alloy stepped tube by experiments and finite element numerical simulation. Anaraki et al. [22] used experimental and numerical simulation methods to study the influence of pulsating pressure on HMGF process. The results show that the proposed pulsating pressure path improves the gas expansion formability and wall thickness distribution along the circumference of the hot metal tube. Maeno et al. [23] studied the HMGF process of an aluminum alloy tube by filling air into the sealed pipe and heating with resistance. Wu et al. [24] studied the deformation process of tubular gas forming under different loading paths at 800 °C. The influence of loading path on forming quality of tubular parts was studied by theoretical analysis, finite element simulation and experiment. Liu et al. [25] studied the influence of axial feed on the deformation behavior of Ti-3Al-2.5V tubular parts, such as thickness distribution, microstructure and mechanical properties. The results show that, with the increase in the feed, the average thinning rate of the bulging area decreases obviously, and the thickness distribution of the bulging parts becomes more uniform. He et al. [26] conducted free expansion tests on an AA6061 extruded tube at different temperatures ranging from 350 °C to 500 °C. The results show that the largest maximum expansion ratio is 86% at 425 °C. Bursting pressure decreases from 4.4 MPa to 1.5 MPa with temperature increasing. The initial fine equiaxial grain grows as temperature increases, which is elongated simultaneously in both axial and hoop directions.

However, many studies mainly focus on the HMGF process on tubular parts with simple variable section, where the structure of aviation integral hollow member is very complicated, and research into the HMGF process of aluminum alloy complex thin-wall hollow member has not been perfected. The microstructure of aluminum alloy under different HMGF process parameters needs further study. Therefore, HMGF process of double wave tube with large deformation volume was studied in this paper. The effects of different temperatures and pressures on the formability of 6063 aluminum alloy tubes were studied. The deformation behavior, microstructure evolution and hot deformation mechanism of the material in HMGF process were analyzed. Finally, the optimal hot metal gas forming process parameters of 6063 aluminum alloy tubes were explored to obtain high precision forming parts.

## 2. Material and Experimental Methods

### 2.1. Material and Objective Part

**Figure 1:**
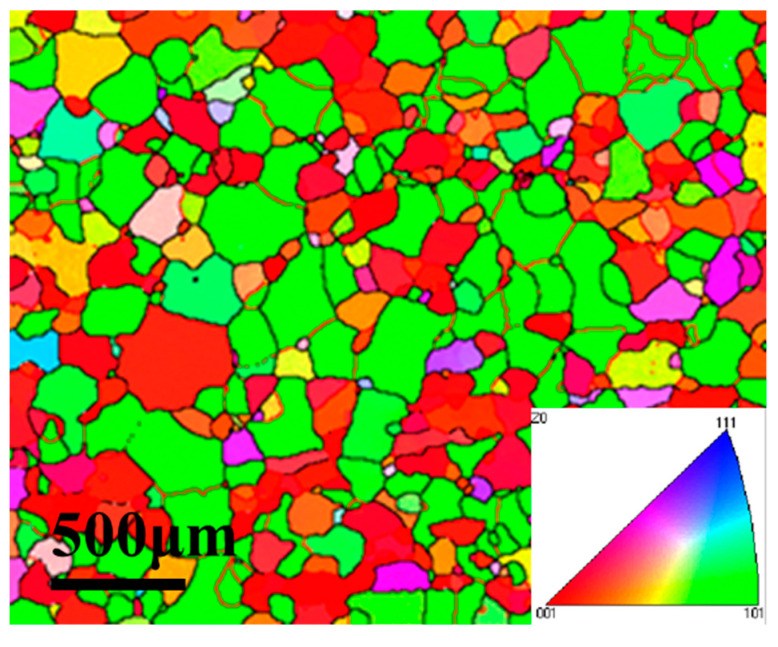
Initial structure of 6063 aluminum alloy tube.

**Figure 2:**
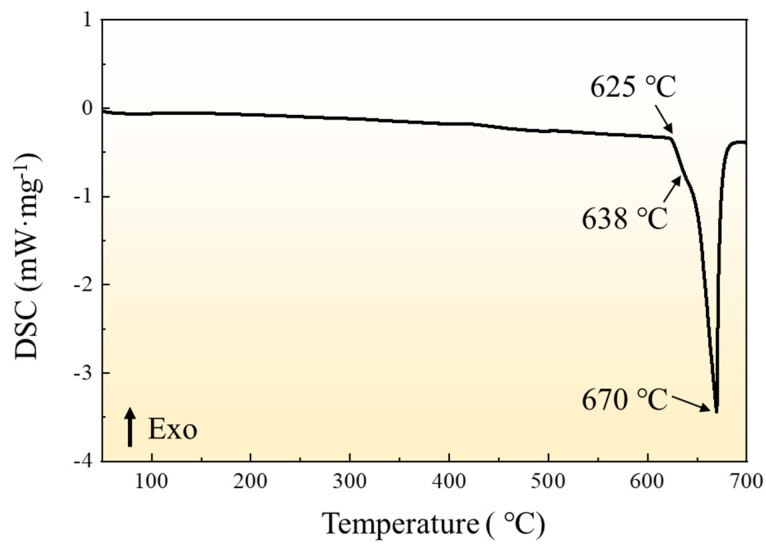
The DSC curve of 6063 aluminum alloy.

Hot extrusion of 6063 aluminum alloy tube was used in the present work. The tubes are purchased from a domestic manufacturer named Shanghai Yifei Metal Co. LTD. The chemical composition was analyzed by Inductively Coupled Plasma Atomic Emission Spectrometer (ICP-AES6300), as shown in Table 1. The initial microstructure of the tube is shown in Figure 1, and the grain size distribution of the hot extrusion tube is uniform. In order to determine the forming temperature of the tube, the melting point of the tube sample was tested by differential thermal analysis (DSC), as shown in Figure 2. The melting point of 6063 tube was 625 °C, and the hot forming temperature should not exceed the melting point.

**Figure 3:**
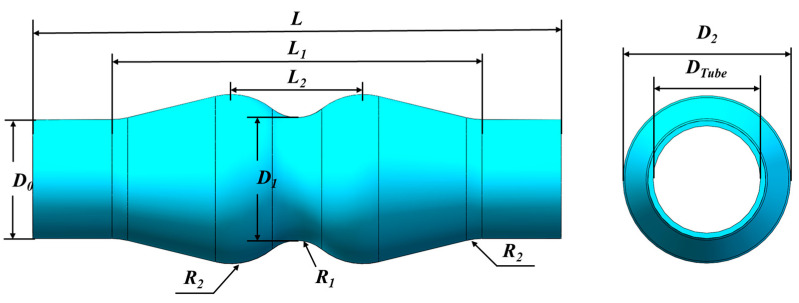
Shape and dimensions of the objective part.

A double wave tube was selected and investigated in this paper, as shown in Figure 3; the specific size is shown in Table 2. The outer diameter of the tube sample is Φ 45 mm, the wall thickness is 2 mm, the required length of the tube sample is calculated as 240 mm and the length of the tube sample is set as 280 mm by increasing the allowance. The deformation area of the target part is 70%. The structure of the target part is complex with double peak and single valley structure.

The section change rate is used to evaluate the difficulty of expansion forming; the higher the section change rate is, the more difficult it is to form. The section change rate is calculated as follows:

(1) $$\eta =\frac{D_{i}-D_{0}}{D_{0}}$$

where $D_{i}$ is the diameter of arbitrary cross section i (perpendicular to the central axis) of the tube after expansion, and $D_{0}$ is the initial diameter of the tube.

The section change rate of the target part at the peak position is 42.2%, which is the largest, and the section change rate at the valley position is 3.2%. It belongs to the large deformation and large section change rate tube, and the forming difficulty is high. At the same time, compared with the single peak parts with the same maximum section change rate, the forming difficulty is higher because the expansion of double peaks is affected by the insufficient feeding in the middle position.

### 2.2. Principle and Equipment of HMGF Process

**Figure 4:**
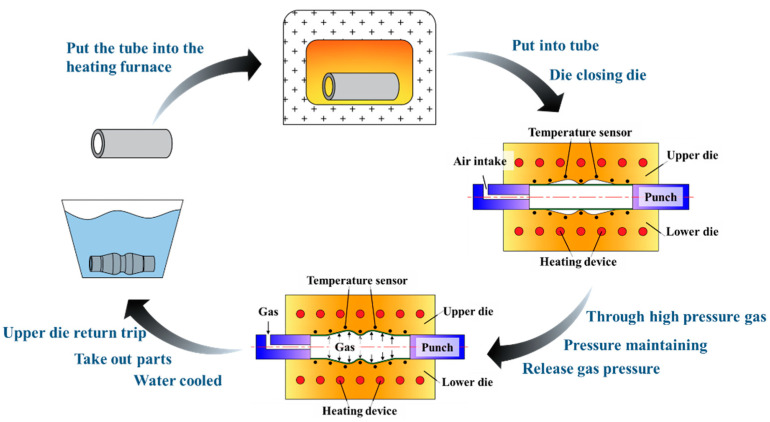
Schematic diagram of hot gas bulging.

The principle of HMGF process is shown in Figure 4. The heated tube sample was put into the pre-heated forming die. The forming die and the tube sample were heated together to the required temperature and stabilized by a heating device. High pressure gas was injected into the tube to make the material expand and deform. After forming, the part was quickly removed and cooled to room temperature. The HMGF process can realize the forming of tubes near the recrystallization temperature, which greatly improves the formability of the parts. Therefore, it is suitable for the forming of a double wave tube with high forming difficulty in this paper.

**Figure 5:**
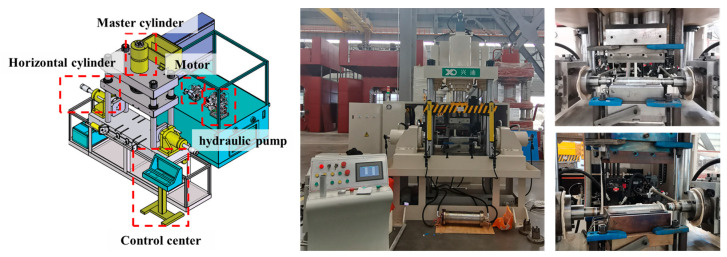
The equipment of HMGF.

The forming equipment was developed according to the principle of HMGF process, as shown in Figure 5. In this paper, nitrogen gas is used, and the highest gas pressure of the equipment is 15 MPa. The upper and lower die are installed with heating tubes for heating. PID temperature controller was used for high temperature control, and the temperature accuracy is ±2 °C. Forming die can be used safely at 500 °C and below. The pressure range is 1~3 MPa [26] and the temperature range is 400~475 °C.

## 3. Effect of Pressure on Formability and Microstructure of 6063 Tube

### 3.1. Effect of Pressure on the Formability of 6063 Tube

**Figure 6:**
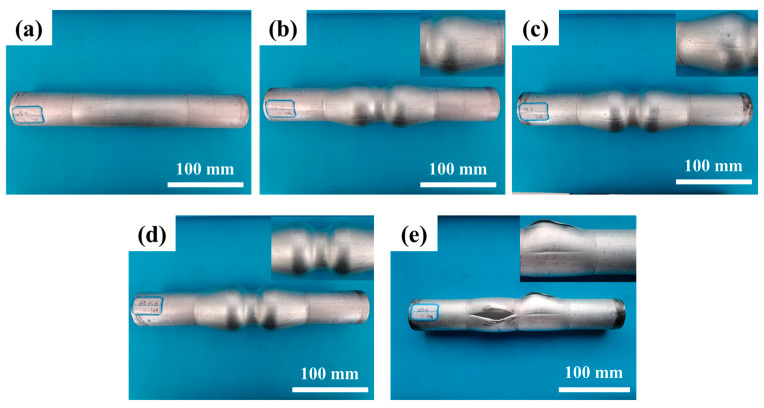
Forming results of parts under different forming pressures at 475 °C: (**a**) 1 MPa; (**b**) 1.5 MPa; (**c**) 2 MPa; (**d**) 2.5 MPa; (**e**) 3 MPa.

The pressure provided by the gas medium during tube forming can be considered to be uniform. Under the constant pressure rate, the greater the pressure, the greater the deformation on the unit area of the tube. HMGF of 6063 tubes was carried out at a forming temperature of 475 °C and forming pressures of 1 MPa, 1.5 MPa, 2 MPa, 2.5 MPa and 3 MPa, respectively. The experimental results are shown in Figure 6. It can be seen from the forming results that when the pressure is less than 2 MPa, the formability of the tube is lower and the expansion height is insufficient. When the pressure is 3 MPa, the stress generated by the pressure loading exceeds the material limit, and the tube has an obvious fracture defect.

The maximum section change rate of the part is an important index to evaluate the forming effect of the expanded tube. The closer the maximum section change rate of the formed part to the target value, the better the forming effect will be. For the convenience of comparison, the maximum relative section change rate $\eta_{max}^{\prime }$ is defined to evaluate the forming capacity of the tube as shown in Equation (2). $\eta_{max}^{\prime }$ is the ratio of the maximum section change rate ($\eta_{max}^{i}$) of the forming tube and the section change rate ($\eta_{max}^{0}=42.2\%$) of the target part. The closer value of $\eta_{max}^{\prime }$ to 100%, the better the forming effect is.

(2) $$\eta_{max}^{\prime }=\frac{\eta_{max}^{i}}{\eta_{max}^{0}}=\frac{\frac{D_{max}^{i}-D_{0}}{D_{0}}}{\frac{D_{max}^{0}-D_{0}}{D_{0}}}=\frac{D_{max}^{i}-D_{0}}{D_{max}^{0}-D_{0}}$$

where $\eta_{max}^{i}$ and $\eta_{max}^{0}$ are the maximum section change rates of the formed part and the target part, respectively. $D_{max}^{i}$ and $D_{max}^{0}$ are the diameters at peak position of the forming part and the target part, respectively.

**Figure 7:**
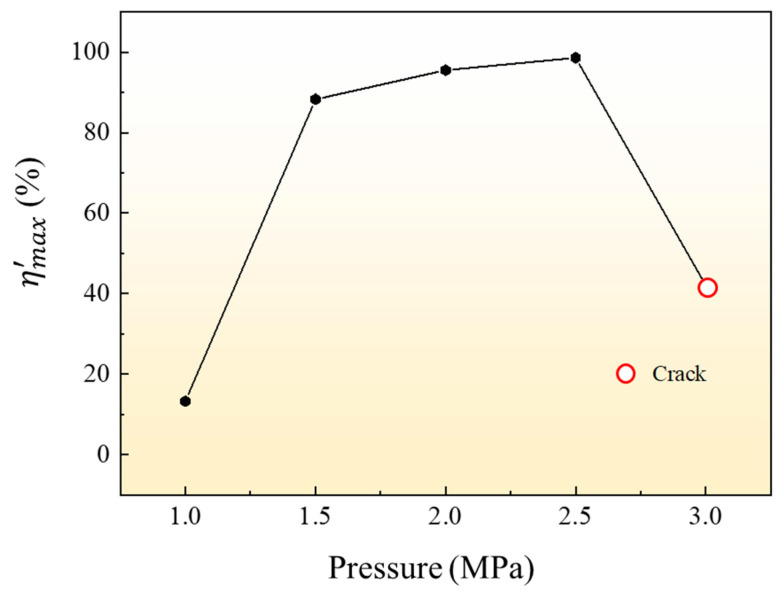
Influence of different pressure on $\eta_{max}^{\prime }$ at 475 °C.

The maximum relative section change rate of the formed parts was measured, and the results were shown in Figure 7. The data when forming pressure is 3 MPa is not desirable due to fracture defect. It can be seen from the results that when the forming pressure is 2 MPa and 2.5 MPa, the maximum relative section change rate of the forming part remains at a high level, and the maximum relative section change rates are 95.5% and 98.6%, respectively.

### 3.2. Effect of Pressure on the Microstructure of 6063 Tube

**Figure 8:**
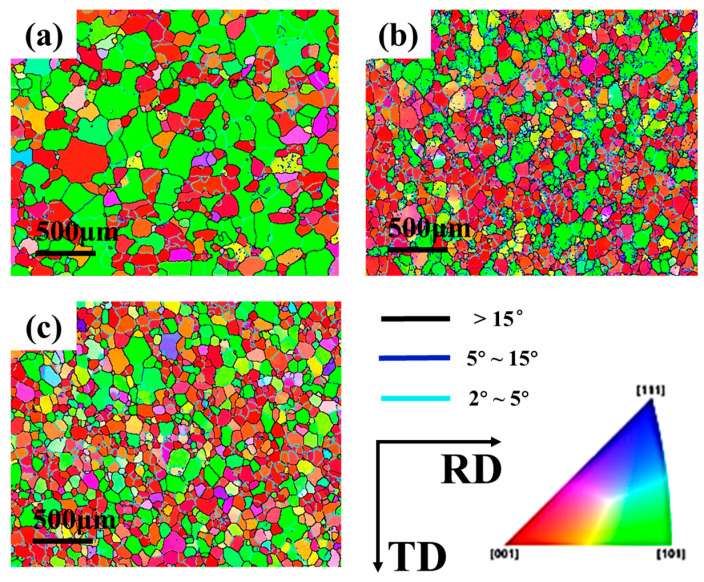
Microstructure morphology of tubes under different pressure: (**a**) original; (**b**) 1 MPa; (**c**) 1.5 MPa.

The effect of pressure on microstructure was analyzed by selecting the tube with a large difference in deformation degree. Therefore, the initial sample and the parts under forming pressure of 1 MPa and 1.5 MPa are selected for EBSD analysis. The sampling position was the peak position of the forming part. The microstructure morphology is shown in Figure 8. Different types of grain boundaries are divided by different colors in the figure where subgrain boundaries of 2°~5° are shown in dark blue, small Angle boundaries of 5°~15° are shown in light blue and large Angle boundaries above 15° are shown in black lines. It can be seen from the results that the internal structure of the initial tube is uniform with an average grain size of 181.01 μm. When the pressure is 1 MPa, the number of large size grains decreases slightly and the average grain size is 119.43 μm. When the pressure of the part is increased to 1.5 MPa, the density of the substructure in the material is decreased obviously, and there are more recrystallized grains with increased size in the grain. The average grain size of the material is 105.19 μm.

**Figure 9:**
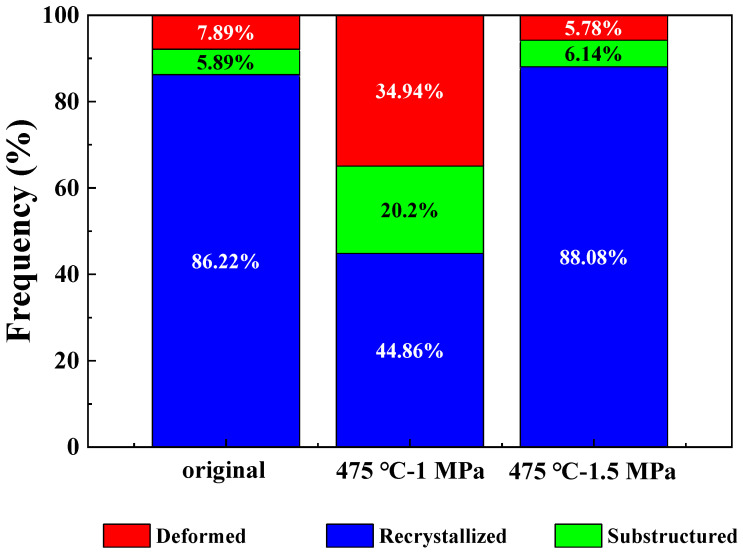
The microstructure of the materials under different pressures at 475 °C.

The microstructure ratio of materials under different bulging pressures was analyzed. As shown in Figure 9, the recrystallization microstructure content of materials under different pressures was different. When the bulging pressure increased to 1.5 MPa, the recrystallization microstructure ratio increased by 96.3% from 44.86% to 88.08%. The proportion of responding organizations decreased from 20.20% to 6.14%. When the strain variable is small, the softening behavior of the material is the result of dynamic recovery and dynamic recrystallization. When the material is deformed at high temperature with large strain, the dynamic recrystallization is the main dynamic softening mechanism [15,27].

## 4. Effect of Temperature on Formability and Microstructure of 6063 Tube

### 4.1. Effect of Temperature on the Formability of 6063 Tube

**Figure 10:**
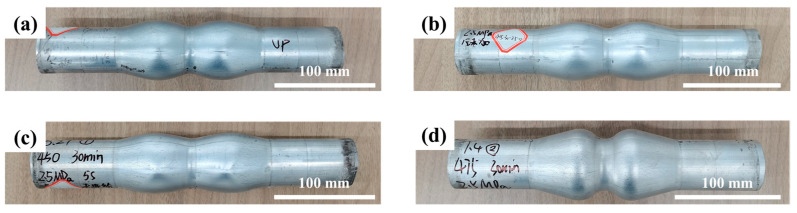
Forming results of parts under different temperatures: (**a**) 400 °C; (**b**) 425 °C; (**c**) 450 °C; (**d**) 475 °C.

The influence of forming temperature on HMGF formability of 6063 aluminum alloy tube was studied. HMGF of 6063 tubes was carried out at a forming pressure of 2.5 MPa and forming temperatures of 400 °C, 425 °C, 450 °C and 475 °C, respectively. The forming results are shown in Figure 10. It can be seen from the results that under the same pressure, the forming effect of the forming parts is obviously improved with the increase in temperature. When the temperature is lower than 475 °C, the formability of the tube is lower and the expansion height is insufficient. The forming effect is better when the forming temperature is 475 °C.

**Figure 11:**
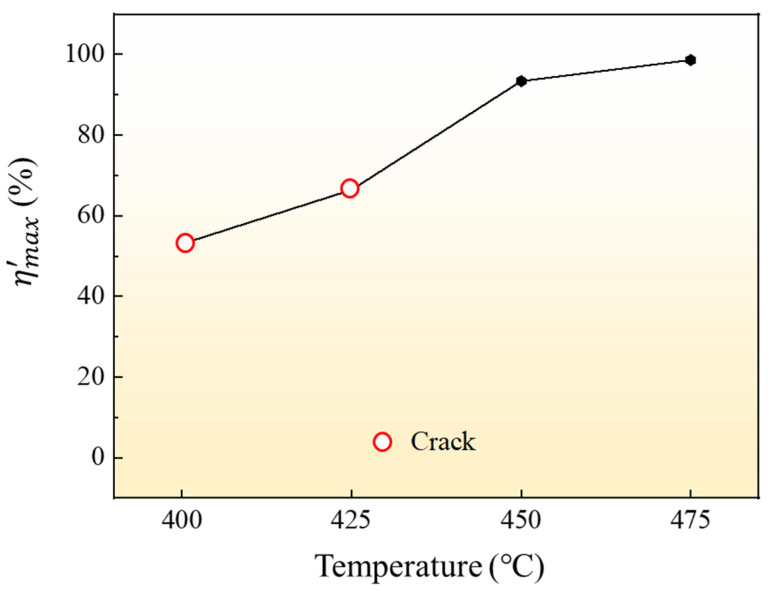
Influence of different temperatures on $\eta_{max}^{\prime }$ at forming pressure 2.5 MPa.

The maximum relative section change rate of the formed parts under different temperatures was measured, and the results were shown in Figure 11. The data when forming temperature is 400 °C and 425 °C are not desirable due to fracture defect. It can be seen from the results that the maximum relative section change rate of the forming parts can reach 93.4% when the forming temperature is 450 °C. With the increase in temperature, the maximum relative section change rate of parts increases, and the maximum relative section change rate of parts increases to 98.6% when the forming temperature is 475 °C.

### 4.2. Effect of Temperature on the Microstructure of 6063 Tube

**Figure 12:**
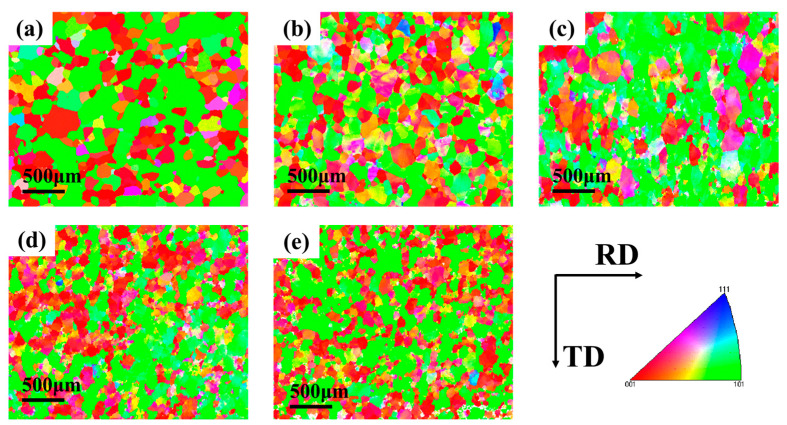
Reverse polar diagrams at different temperatures: (**a**) original; (**b**) 400 °C; (**c**) 425 °C; (**d**) 450 °C; (**e**) 475 °C.

**Figure 13:**
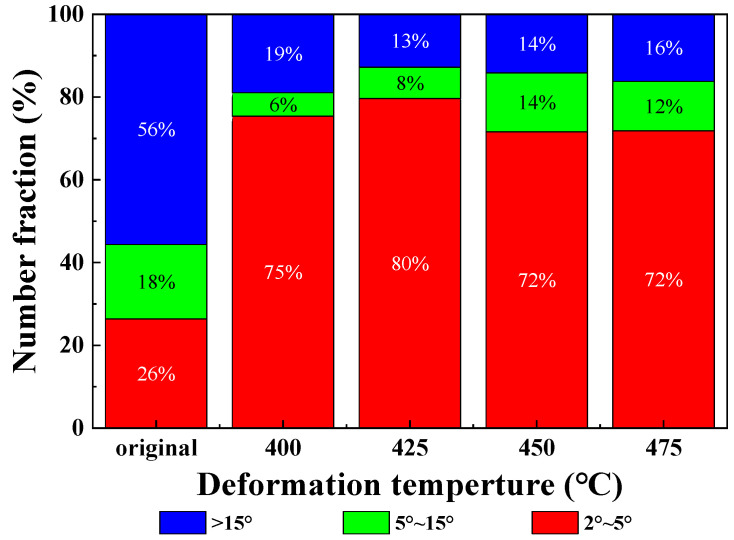
Reverse polar diagrams at different temperatures.

The effect of temperature on microstructure was analyzed by selecting the tube with different forming temperatures under forming pressure of 2.5 MPa. The sampling position was also the peak position of the forming part. The microstructure morphology was shown in Figure 12. It can be seen from the results that the higher the deformation temperature, the finer the grain size and the more uniform the orientation distribution under the same loading path. The comparison of grain boundary angles distribution at different temperatures was shown in Figure 13. Compared with the initial sample, the proportion of small Angle grain boundaries in the alloy after hot deformation is increased, and the proportion of 2°~5° grain boundaries is larger, indicating that the process of nucleation and growth of subcrystal is generated during hot deformation.

**Figure 14:**
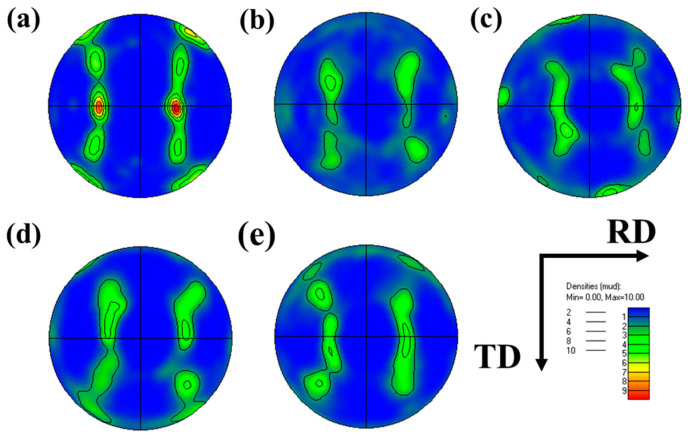
Texture types at different temperatures: (**a**) original; (**b**) 400 °C; (**c**) 425 °C; (**d**) 450 °C; (**e**) 475 °C.

**Figure 15:**
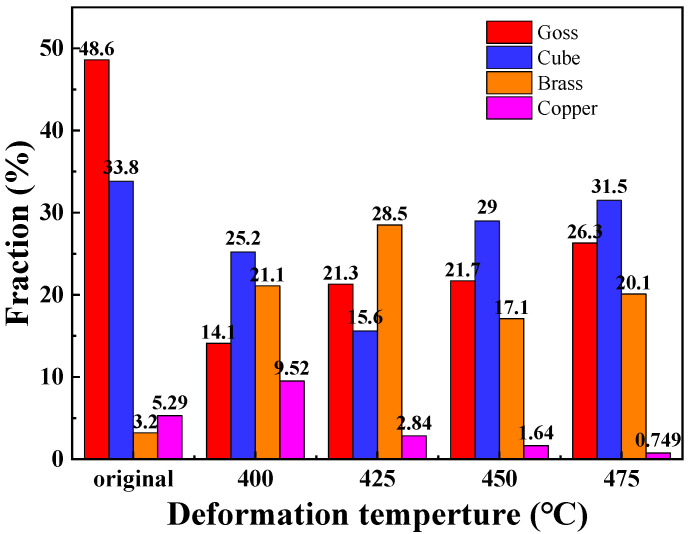
Comparison of texture contents at different temperatures.

As a typical Face Centered Cubic metal, Cube texture {001}<100>, Copper texture {112}<111>, Brass texture {011}<211> and Goss texture {011}<100> are the main deformation textures of 6063 aluminum alloy [28]. The {111} pole diagram and texture content diagram at different forming temperatures were shown in Figure 14 and Figure 15, respectively. Goss texture and Cube texture are typical recrystallization textures of aluminum alloys, and their volume fractions reflect the degree of recrystallization of materials [29]. The initial sample was the seamless aluminum alloy tube made by hot extrusion with high recrystallization degree, so the Goss texture and Cube texture account for a large proportion. The volume fraction of Goss texture and Cube texture increases with the increase in deformation temperature in the process of hot deformation, and the content of both textures is 57.8% at 475 °C. With the increase of deformation temperature, the volume fraction of Copper texture shows a decreasing trend, which is 9.52% at 400 °C but 0.75% at 475 °C. After hot deformation, the content of Brass texture in the material is significantly increased compared with that in the initial tube, and the content is all above 17%.

**Figure 16:**
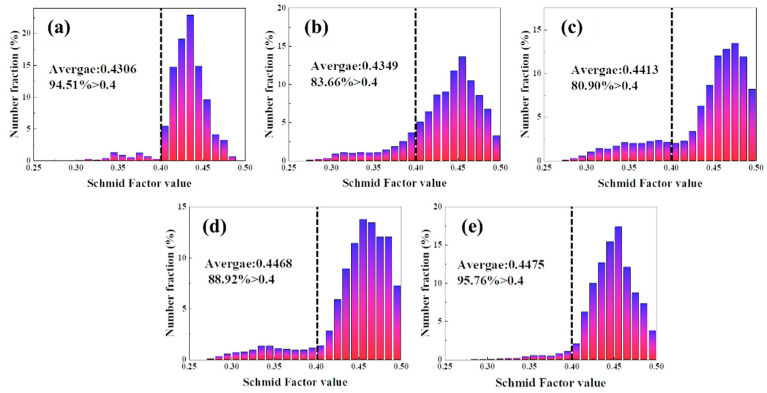
Schmid Factor distribution at different temperatures: (**a**)original; (**b**) 400 °C; (**c**) 425 °C; (**d**) 450 °C; (**e**) 475 °C.

The activation of slip system is related to the Schmid Factor value of materials [30]. For aluminum alloy with multiple sliding systems, the closer the maximum Schmid factor is to 0.5, the greater the possibility of material sliding system activation, and the lower the yield stress of the material [31]. Figure 16 shows the mean value of Schmid Factor at different deformation temperatures. The average Schmid Factor value of the initial tube is 0.4306 and the proportion of Schmidt factor value greater than 0.4 accounted for 94.51%. When the deformation temperature was 400 °C, the average Schmid Factor value of the tube is 0.4349, and the proportion of Schmidt factor value greater than 0.4 is 83.66%. With the increase in deformation temperature, the average Schmid Factor value of the material increases gradually. When the deformation temperature was 475 °C, the average Schmid Factor value is 0.4475, and the proportion of Schmidt factor value greater than 0.4 is as high as 95.76%.

**Figure 17:**
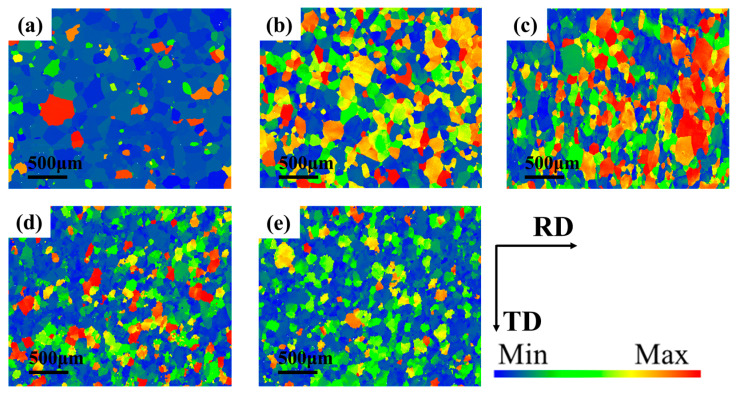
Taylor Factor distribution at different temperatures: (**a**) original; (**b**) 400 °C; (**c**) 425 °C; (**d**) 450 °C; (**e**) 475 °C.

The plastic deformation ability of a single grain can be expressed by the Taylor Factor, and the value of the Taylor Factor is negatively correlated with the plastic deformation ability of the material [32]. Taylor Factor distribution of 6063 aluminum alloy at different deformation temperatures was shown in Figure 17. The smaller the difference of Taylor Factor values among grains, the better the coordination deformation ability between them. Most of the grains inside the original tube are blue, and very few grains are red. When the deformation temperature was 400 °C and 425 °C, most of the grains in the tube are difficult to deform showing in red. With the increase of deformation temperature, the proportion of blue grains increases, and the deformation coordination between grains is enhanced. During HMGF process, 6063 aluminum alloy will not only undergo work hardening but also dynamic softening [33]. Improvement of deformation temperature is conducive to the dynamic recrystallization of the tube, resulting in softening of the material and enhanced deformation uniformity between grains, so that the formability of the material is improved.

## 5. Optimum HMGF Process Parameters of 6063 Aluminum Alloy Tube

**Figure 18:**
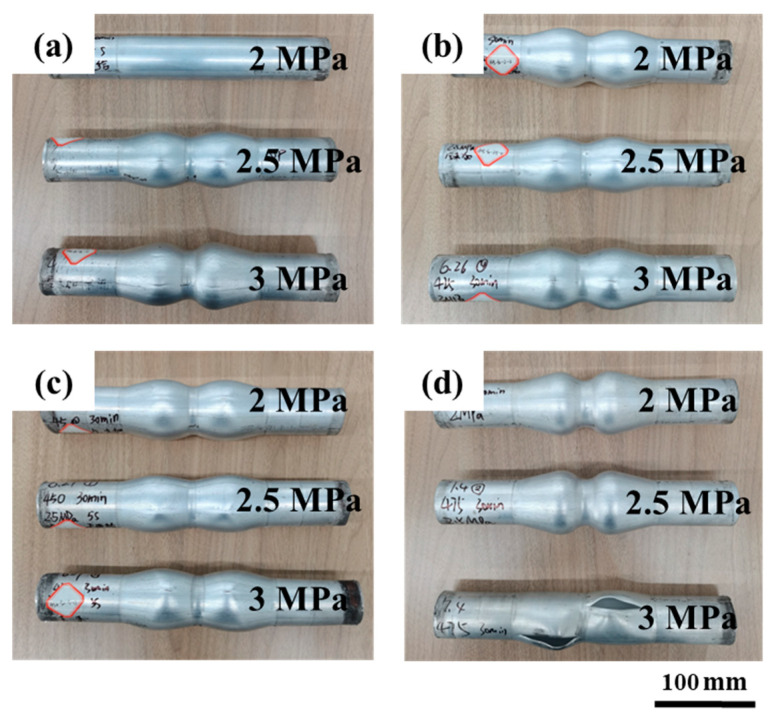
The experimental results of HMGF of 6063 aluminum alloy tube: (**a**) 400 °C; (**b**) 425 °C; (**c**) 450 °C; (**d**) 475 °C.

According to the above results, the forming capacity of the forming parts is affected by the combination of forming temperature and forming pressure. Different forming temperatures and forming pressures were designed to obtain the best forming process parameters. The forming temperature was 400~475 °C and forming pressure was 2~3 MPa. HMGF of 6063 tubes were carried out under the above process parameters and the experimental results were shown in Figure 18. It can be seen from the results that when the temperature is low and the pressure is low (forming temperature is 400 °C, forming pressure is 2 MPa), the parts are almost without deformation. When the temperature is higher and the pressure is higher (forming temperature is 475 °C, forming pressure is 3 MPa), the part has a serious cracking defect. The forming results show that the forming capacity of the formed part is affected by the interaction between forming temperature and forming pressure. Therefore, the maximum relative section change rate was measured to find the optimal process parameters.

**Figure 19:**
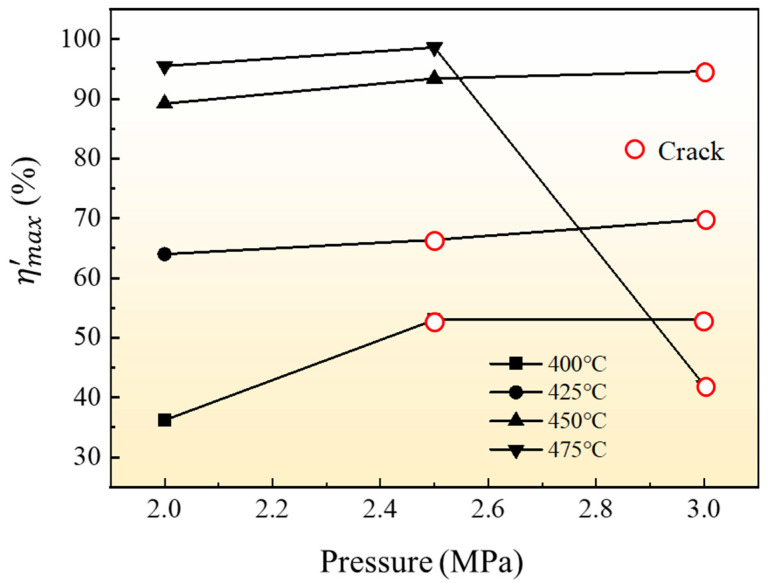
$\eta_{max}^{\prime }$ of 6063 aluminum alloy tube at different forming parameters.

The results of the maximum relative section change rate $\eta_{max}^{\prime }$ of all the formed parts in Figure 18 are shown in Figure 19. It can be seen from the results that the forming temperature has a greater effect on the expansion result of the tube than the forming pressure, and the maximum relative section change rate of the parts increases obviously when the forming temperature increases. When the forming pressure is 2 MPa, the maximum relative section change rate of the parts formed at 400 °C is only 36.2%, and the maximum relative section change rate increases to 95.5% when the temperature rises to 475 °C. When the forming temperature is 400 °C, the maximum relative section change rate of parts with increasing forming pressure does not increase much and rupture defects are easy to occur. When the forming temperature is 475 °C and the forming pressure is 2.5 MPa, the maximum relative section change rate of the forming tube is the highest, which is 98.6% (the maximum expansion ratio is 41.6%). To sum up, the optimum HMGF process parameters of 6063 aluminum alloy tubes are 475 °C with pressure of 2.5 MPa.

## 6. Conclusions

In this paper, the hot metal gas forming process of 6063 aluminum alloy tubes under different temperatures and pressures was carried out, and the formability and microstructure of the alloy were analyzed. The main research conclusions are as follows:

1. The maximum relative section change rate increases with the increase in pressure. When the strain variable is small, the softening behavior of the material is the result of dynamic recovery and dynamic recrystallization. When the material is deformed at high temperature with large strain, the dynamic recrystallization is the main dynamic softening mechanism.
2. Improvement of deformation temperature is conducive to the dynamic recrystallization of the tube, resulting in softening of the material and enhanced deformation uniformity between grains, so that the formability of the material is improved.
3. The forming capacity of the forming parts is affected by the interaction between forming temperature and forming pressure. The forming temperature has a greater effect on the expansion result of the tube than the forming pressure. The optimum HMGF process parameters of 6063 aluminum alloy tubes are 475 °C with pressure of 2.5 MPa.

## Figures and Tables

**Table 1:** Chemical composition of 6063 aluminum alloy w/%.

Mg	Si	Cr	Mn	Fe	Cu	Zn	Al
0.58	0.46	<0.03	0.06	0.19	0.03	<0.03	Balance

**Table 2:** The dimensions of the objective part.

D_Tube_ (mm)	D_0_ (mm)	D_1_ (mm)	D_2_ (mm)	L (mm)	L_1_ (mm)	L_2_ (mm)	R_1_ (mm)	R_2_ (mm)
41	45	46.45	64	200	140	50	15	25

## Data Availability

Not applicable.
